# Giant cell tumour of soft tissue—a rare presentation of a common pathology

**DOI:** 10.1259/bjrcr.20200012

**Published:** 2020-05-27

**Authors:** Annu Chopra, Matthew Kinsella, Sara Edwards, Ian Smith, Philip Robinson

**Affiliations:** 1 Northern General Hospital, Herries Road, Sheffield, UK; 2 Dermatopathologist St.James's University Hospital, Leeds, LS9 7TF, UK; 3 Plastic Surgeon St.James's University Hospital, Leeds, LS9 7TF, UK; 4 Radiology Department, Leeds Teaching Hospitals, Chapel Allerton Hospital, Leeds, LS7 4SA, UK; 5 University of Leeds and NHIR Leeds Biomedical Research Unit, Chapel Allerton Hospital, Leeds, UK

## Abstract

We present the case of a giant cell tumour of soft tissue (GCT-ST) presenting as a slow-growing paraspinal mass. Imaging investigations revealed a well-circumscribed subcutaneous lesion containing fluid–fluid levels and an internal solid nodule. The imaging findings resulted in only a tentative differential which included haematoma or complex epidermoid cyst but failed to provide a definitive diagnosis. The patient underwent an image-guided biopsy from which a histopathological diagnosis of a GCT-ST was made. GCT-ST is a primary soft tissue neoplasm that is clinically and histologically similar to giant cell tumour of bone. Given its rare occurrence, there is very little published literature on the characteristic imaging findings of GCT-ST to help with its diagnosis which is usually only made histologically. The aim of this case report is to highlight our specific imaging findings and add to the limited pre-existing imaging data on GCT-ST.

## Introduction

Giant cell tumour of soft tissues (GCT-ST), also known as “giant cell tumour of low malignant potential“ is a rare presentation of the relatively common pathological entity of giant cell tumours. It is clinically and histologically similar to giant cell tumours of bone, classically presenting as painless masses within the extremities and the trunk. Given its rare occurrence, very little can be found in the literature regarding the imaging characteristics of GCT-ST other than that presented in a few case reports. We report a case of a paraspinal GCT-ST and show how the imaging findings correlate with the histological features of GCT of bone.

## Case report

**Figure 1.**
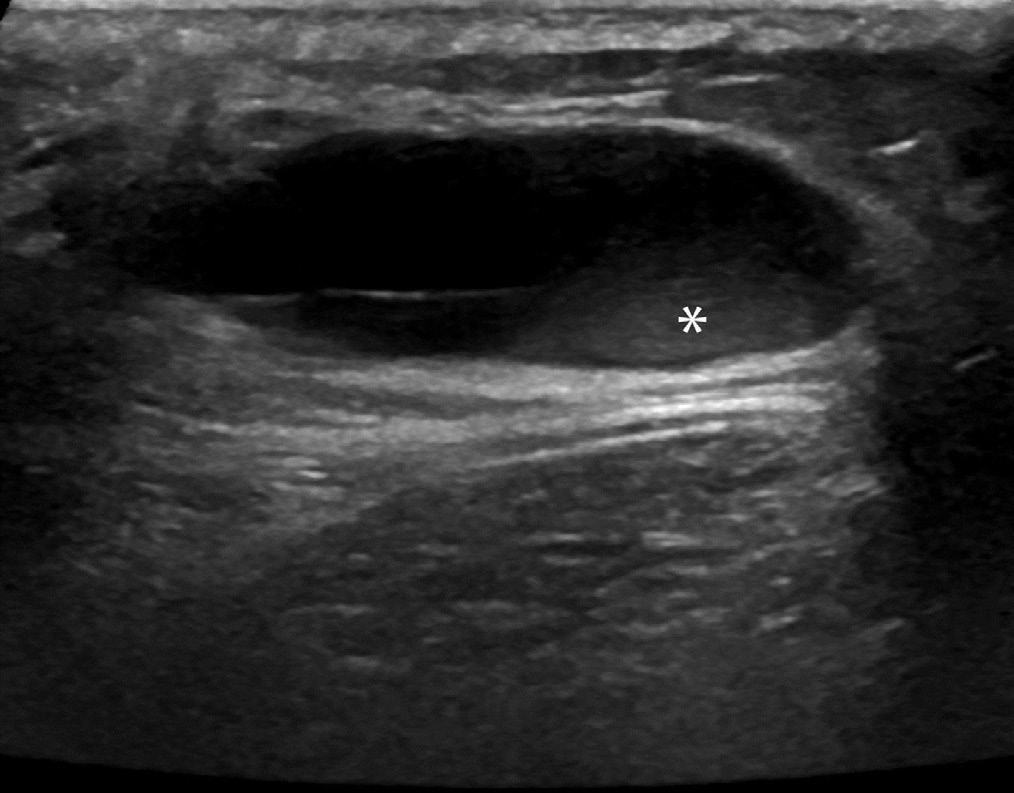
Transverse sonographic image of the upper back shows a predominantly anechoic subcutaneous lesion with posterior acoustic enhancement containing a small soft tissue nodular component (*).

**Figure 2.**
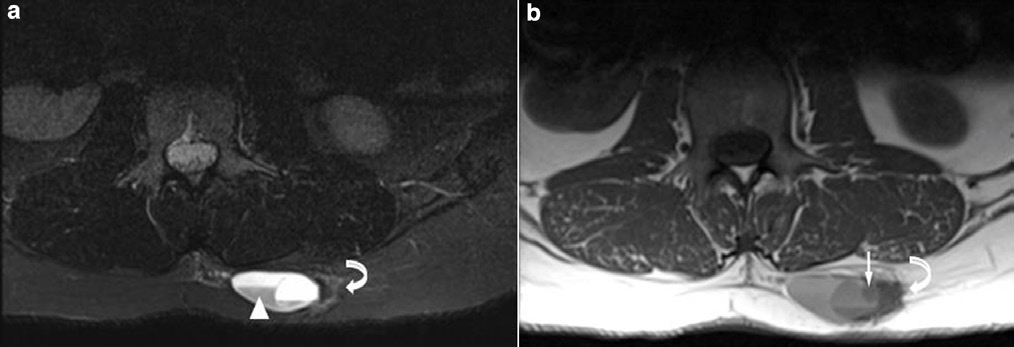
(a) Axial T2 STIR & (b) Axial T1 MR images show a well-defined subcutaneous lesion containing fluid–fluid levels (white arrowhead) and an internal solid nodule (white arrow) with ill-defined low signal change in the adjacent subcutaneous fat on the lateral margin (curved arrow). STIR, short tau inversion recovery.

**Figure 3.**
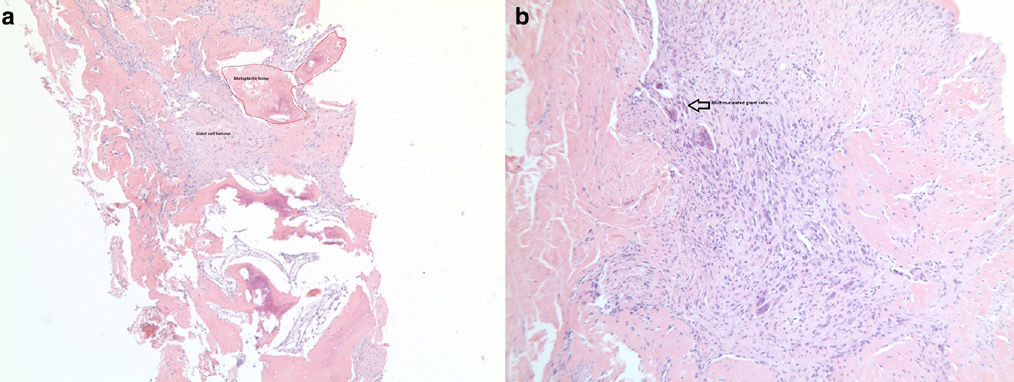
Histopathology slide from the soft tissue biopsy shows 3a metaplasia around the soft tissue GCT and 3b multinucleated giant cells within the soft tissue GCT. GCT, giant cell tumour.

**Figure 4.**
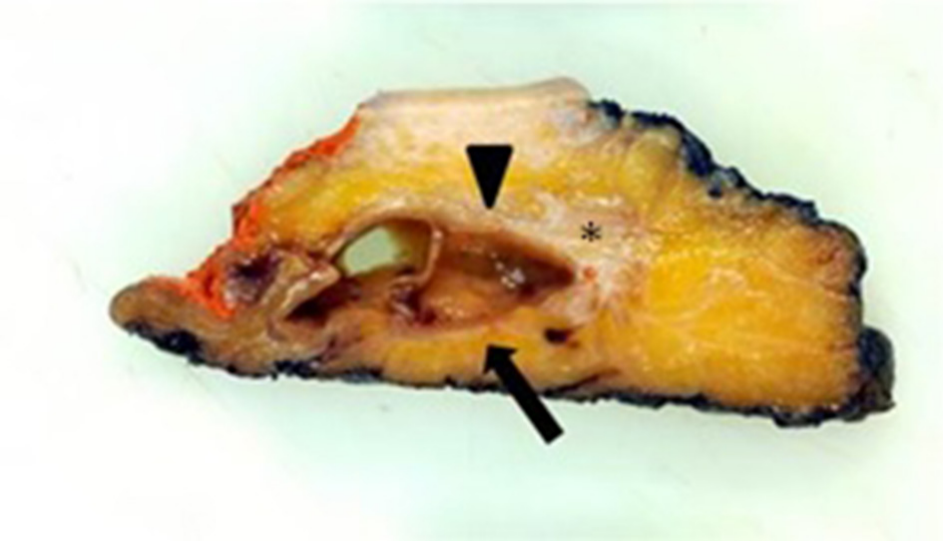
Section through the surgical specimen shows a cystic mass (arrowhead) with an internal soft tissue nodule (arrow) and lateral fat change (*).

A fit and well 30-year-old female presented to her primary care practitioner with a 12 month history of a slow-growing left paraspinal lump in the absence of any trauma. She had an initial ultrasound scan (Figure 1) which showed a subcutaneous and predominantly cystic mass containing a solid nodule. The patient was referred for an MRI for further characterisation and this revealed a relatively well-circumscribed subcutaneous lesion containing fluid–fluid levels and an internal solid nodule (Figure 2a and b) with some ill-defined low *T*_1_W and *T*_2_W signal change within the fat along the lateral margin (curved arrow). The imaging findings were not characteristic of any specific pathology in this location and a differential diagnosis of possible haematoma or complex epidermoid cyst was given. The patient was referred to the local sarcoma service and had a subsequent image-guided biopsy from which a histological diagnosis of GCT-ST was made (Figure 3). Following on from this, the patient underwent complete surgical resection of the mass with histologically proven clear resection margins (Figure 4).

## Discussion

GCT-ST was first described by Salm and Sissons in 1972 where it was initially considered under the umbrella term of “malignant giant cell tumour of soft parts“.^1^ However, subsequent detailed pathological analysis of these tumours found that they lack cytologic atypia, even in the presence of increased mitotic activity and vascular invasion and thus they were reclassified by Folpe et al as “giant cell tumours of low malignant potential“.^2^ This is a primary soft tissue neoplasm that is clinically and histologically similar to giant cell tumour of bone because it demonstrates multinodular aggregates of round to spindle-shaped neoplastic cells, admixed with numerous, uniformly scattered osteoclast-like multinucleated giant cells (Figure 3).^3^ It occurs predominantly in the fifth decade of life and has no sex predilection. These tumours usually develop in the superficial soft tissues of the extremities and less frequently within the trunk and head & neck.^4^ GCT-ST is expected to run a benign course as the malignant potential of these tumours, as determined by their histological characteristics is considered to be low and metastases are very rare.^5^ Complete surgical excision with clear resection margins is expected to be curative.

GCT-ST is relatively rare with only a few reported cases,^4,6,7^ and thus characteristic radiological findings for this tumour have not been documented. In the present case the ultrasound image (Figure 1) shows a well-circumscribed subcutaneous lesion which is predominantly cystic but also has a small echogenic solid component correlating with the gross histopathology specimen (Figure 4). The MRI also reveals a complex cystic lesion containing fluid–fluid levels as well as internal solid areas (Figure 2). The cystic element of giant cell tumours is due to the development of aneurysmal bone cyst components which can haemorrhage leading to fluid–fluid levels as in the present case. This is recognised in 14% of giant cell tumours as well being the commonest lesion associated with secondary aneurysmal bone cyst (ABC) formation.^8^ The ill-defined low signal change within the fat lateral to the lesion corresponded to an area of histiocytic infiltration and fibrosis within the adipose tissue consistent with reactive changes, presumably to possible previous extravasation of fluid into the adjacent fat. With the benefit of hindsight it can be appreciated that the imaging findings do resemble the features seen with GCT of bone and secondary ABC development. However, given the atypical location and rarity of this tumour the correct diagnosis was not considered, even as part of the differential in this case. This case report highlights the imaging findings of benign GCT-ST, which are similar to another published case report^7^ also presenting ultrasound and MRI evaluation. In that case, the mass was also subcutaneous with two well-defined simple cystic areas demonstrated on ultrasound and MRI, but showed no evidence of ABC development. Ultrasound features were very similar to the current case but MRI features differed with better demonstration of fluid–fluid levels and multiloculation seen in the current case. Two other case reports showed quite different ultrasound and MRI features and are summarised in Table 1^9,10^. In conclusion, we present imaging findings for a rare presentation of benign GCT-ST which should be considered in the differential diagnosis of subcutaneous complex cystic masses particularly if fluid–fluid levels are seen suggesting secondary ABC development.

**Table 1.** Summary of previous case reports

Authors	Clinical	Ultrasound	MRI
Dodd et al 2004^9^	50-year-old male with several months of swelling and pain over lateral left leg. Initially attributed to a minor trauma by patient and subsequently diagnosed as a haematoma and ‘lanced’ Oozing and bleeding post procedure so sought treatment at another institute were a biopsy was performed	Not performed	6 cm solid fungating lesion involving the skin and subcutaneous tissue with poorly defined margins Isointense to muscle on T1, hyperintense signal on STIR, and focal areas of low signal on all sequences. Diffuse enhancement post gadolinium.
Sang et al 2008^7^	23 year old females with a palpable mass in her left thigh that had been present for 1 year The mass had grown in size over a 2 to 3 month period Non-tender and a little warm to palpation	5.5 × 4.8 x 2.4 cm cystic lesion containing a solid nodule and a smaller daughter cyst The cysts were mainly hypoechoic with some dependent debris The nodule was hyperechoic and displayed no doppler flow.	The cysts displayed high T2 signal while the solid nodule displayed low T2 signal There was a low signal rim around the lesion on all sequences. Enhancement of the solid nodule and cyst walls were demonstrated post gadolinium.
Ratebet al 2017^10^	44 year old females with a 3 year history of vague pain in the right thigh. No history of trauma 7 x 4 cm non-tender mass above the right knee	Heterogenous mass with a central hypoechoic area.	7.2 × 5.3 x 2.5 cm mass within rectus femoris Low signal on T1 Hyperintense on T2 with focal low signal areas. The majority of the lesion enhanced post gadolinium.

STIR, short tau inversion recovery.

## Learning points

- GCT-ST are rare benign tumours most commonly found in the superficial soft tissues of the extremities.
- GCT-ST should be considered in the differential diagnosis of a subcutaneous complex cystic mass on ultrasound or MRI.
- The presence of fluid–fluid levels within such a mass is another helpful diagnostic clue as it is suggestive of secondary ABC formation.
